# Beta and gamma human papillomaviruses in anal and genital sites among men: prevalence and determinants

**DOI:** 10.1038/s41598-018-26589-w

**Published:** 2018-05-29

**Authors:** Vitaly Smelov, Richard Muwonge, Olga Sokolova, Sandrine McKay-Chopin, Carina Eklund, Boris Komyakov, Tarik Gheit

**Affiliations:** 1 Prevention and Implementation Group, International Agency for Research on Cancer, World Health Organization, Lyon, France; 2 Infections and Cancer Biology Group, International Agency for Research on Cancer, World Health Organization, Lyon, France; 3 Screening Group, International Agency for Research on Cancer, World Health Organization, Lyon, France; 4 Department of Urology, North-Western State Medical University named after I.I. Mechnikov, St. Petersburg, Russia; 5 Faculty of Medicine, St. Petersburg State University, St. Petersburg, Russia; 6 Clinical Infectious Diseases Hospital named after S.P. Botkin, St. Petersburg, Russia; 7 Department of Laboratory Medicine, Karolinska Institutet, Stockholm, Sweden

## Abstract

Data regarding the anogenital distribution of and type-specific concordance for cutaneous β- and γ-HPV types in men who have sex with women is limited and geographically narrow. Knowledge of determinants of anogenital detection of cutaneous HPV types in different regions is needed for better understanding of the natural history and transmission dynamics of HPV, and its potential role in the development of anogenital diseases. Genital and anal canal samples obtained from 554 Russian men were screened for 43 β-HPVs and 29 γ-HPVs, using a multiplex PCR combined with Luminex technology. Both β- and γ-HPVs were more prevalent in the anal (22.8% and 14.1%) samples than in the genital (16.8% and 12.3%) samples. Low overall and type-specific concordance for β-HPVs (3.5% and 1.1%) and γ-HPVs (1.3% and 0.6%) were observed between genital and anal samples. HIV-positive men had higher anal β- (crude OR = 12.2, 95% CI: 5.3–28.1) and γ-HPV (crude OR = 7.2, 95% CI: 3.3–15.4) prevalence than HIV-negative men. Due to the lack of genital samples from the HIV-positive men, no comparison was possible for HIV status in genital samples. The lack of type-specific positive concordance between genital and anal sites for cutaneous β- and γ-HPV types in heterosexual men posits the needs for further studies on transmission routes to discriminate between contamination and true HPV infection. HIV-positive status may favor the anal acquisition or modify the natural history of cutaneous HPV types.

## Introduction

Human papillomavirus (HPV) is one of the most common sexually transmitted infections (STI) worldwide^1^. Currently, the International HPV Reference Center lists 210 HPV types^2^. The majority of HPVs belongs to the *Alphapapillomavirus* (α), *Betapapillomavirus* (β), and *Gammapapillomavirus* (γ) genera^3^. HPV infections in humans are categorized as mucosal or cutaneous based on their epithelial tropism^4^. So far, 12 mucosal high-risk α-HPVs have been associated with the development of anogenital malignancies and classified by the WHO/International Agency for Research on Cancer as oncogenic to humans^5^.

The oncogenic potential of other genera has not been fully established but rather proposed or speculated in skin cancer for β-HPVs^6–9^ or γ-HPVs^10,11^, respectively. In fact, cutaneous β- and γ-HPVs can be ubiquitously found in swabs of normal skin^12,13^, eyebrow hair samples^13^, in the oral cavity^14,15^ and gargles^16^, nostril^16^ and oesophageal^17^ mucosal samples, and even in faeces, as a result of infecting or passing through the entire digestive system^18^. Additionally, recent studies confirmed the anogenital presence of β- or γ-HPVs on the genitalia^19–21^ and in the anal canal in men^22^. Both β- and γ-HPVs have been detected on the surfaces of male genital lesion samples^19,20,23^ and in vulvar squamous cell carcinoma^24^. Additionally, β-HPVs were also found in penile and anal intraepithelial neoplasia diagnosed in HIV-positive men who have sex with men (MSM), supporting the role of cutaneous HPVs in some cases of neoplasia development^25^. However, most studies on genital HPV infection have examined only α-HPV types^20^.

The presence of β- and γ-HPVs in the anal canal of men has been established^21,26–29^ but the determinants of the anogenital presence of HPVs in males is largely based on geographically-concentrated populations of HIV-positive and HIV-negative MSM^26–28^. It is not clear how cutaneous HPVs are transmitted into the anal canal, i.e., if it can be due to sexual behavior or passing through the entire digestive system. A possible commensal colonization of the anogenital skin with cutaneous HPVs in HIV-positive MSM was posited^21,25^. Moreover, the risk of β-HPV infection in the anogenital region in men who have sex with women (MSW) may occur through direct skin contact^20,30^. Supportively, the first report on sequencing previously unclassified β- and γ-HPVs in the anal canal of men who have sex with women (MSW) from the HIM Study has suggested that other forms of transmission apart from penile-anal intercourse may exist^29^. However, these transmission hypotheses have yet to be fully established.

Studies on genital-anal type-specific positive concordance of cutaneous HPVs involving MSW are nearly absent and represented with the recent report from the HIM Study on the diversity of β-HPV types^21^. To further this etiological knowledge and better understand transmission dynamics of HPV and its role in the development of anogenital diseases, more evidence must be gathered to establish the spectrum of cutaneous HPVs in the anogenital area among diverse population groups worldwide. The aim of the current study is to analyse the prevalence, type-specific positive concordance and determinants for the presence of genital and anal β- and γ-HPVs in a large cohort of MSW from the Eastern hemisphere.

## Results

The characteristics of the remaining 554 (97.7%) men are presented in Table 1. Of those analysed, 31 (5.6%) men were HIV-positive. Genital *C*. *trachomatis* infections was detected in 31 (5.7%) MSW. Up to one third of men (n = 153) reported having Chlamydia infections in the past. Over half of the men (56.3%) had their sexual debut when they were younger than 18 years old. Less than half of the men (47.2%) reported having more than 20 life-time sex partners.

**Table 1:** Characteristics of participants.

Characteristics	Number	Percentage
Individuals assessed	554	
Entry age (years)		
<25	102	19.8
25–29	126	24.5
30–34	134	26.0
35–39	72	14.0
40–44	41	8.0
45+	40	7.8
Age of sex debut		
<18	291	56.3
18+	226	43.7
No. of life-time sex partners		
1–9	149	28.9
10–19	123	23.9
20+	243	47.2
Detected genital *C*. *trachomatis*		
Negative	510	94.3
Positive	31	5.7
Self-reported genital *C*. *trachomatis* in the past		
No	361	70.2
Yes	153	29.8
HIV status		
Negative	523	94.4
Positive	31	5.6

HIV: human immune-deficiency virus.

All samples included were successfully typed for β- and γ-HPVs. β-HPV prevalence was 33.0% (n = 122) for either genital or anal samples, 16.8% (n = 76) for genital and 22.8% (n = 107) for anal sites (Table 2). Values for γ-HPV overall, genital and anal prevalence were 24.3% (n = 75), 12.3% (n = 47) and 14.1% (n = 64), (Table 3). The β-HPV-1, -2, -3 and -5 species and the γ-HPV-1, -3, -7, -10 and -12 species detected were more prevalent in the anal than genital samples (Tables 2 and 3).

**Table 2:** Positivity of cutaneous beta HPV types in the genital and anal samples among men who have sex with women (MSW).

HPV type	Sample site								
	Genital Number (%)		Anal Number (%)		Among those with both genital and anal samples tested				
					Positive on either site Number (%)		Positive on both sites Number (%)		Kappa
Individuals assessed	453		470		370		370		
Any HPV type	76	(16.8)	107	(22.8)	122	(33.0)	13	(3.5)	0.01
HPV 5	9	(2.0)	10	(2.1)	12	(3.2)	0	(0.0)	−0.01
HPV 8	0	(0.0)	8	(1.7)	5	(1.4)	0	(0.0)	0.00
HPV 9	2	(0.4)	2	(0.4)	4	(1.1)	0	(0.0)	−0.01
HPV 12	2	(0.4)	10	(2.1)	5	(1.4)	0	(0.0)	−0.01
HPV 14	3	(0.7)	3	(0.6)	6	(1.6)	0	(0.0)	−0.01
HPV 15	2	(0.4)	4	(0.9)	5	(1.4)	0	(0.0)	−0.01
HPV 17	1	(0.2)	5	(1.1)	5	(1.4)	0	(0.0)	0.00
HPV 19	1	(0.2)	3	(0.6)	1	(0.3)	1	(0.3)	1.00
HPV 21	0	(0.0)	2	(0.4)	1	(0.3)	0	(0.0)	0.00
HPV 22	11	(2.4)	14	(3.0)	14	(3.8)	0	(0.0)	−0.02
HPV 23	10	(2.2)	6	(1.3)	14	(3.8)	0	(0.0)	−0.02
HPV 24	1	(0.2)	2	(0.4)	1	(0.3)	0	(0.0)	0.00
HPV 36	0	(0.0)	4	(0.9)	0	(0.0)	0	(0.0)	—
HPV 37	1	(0.2)	1	(0.2)	1	(0.3)	0	(0.0)	0.00
HPV 38	10	(2.2)	9	(1.9)	5	(1.4)	1	(0.3)	0.33
HPV 47	1	(0.2)	1	(0.2)	1	(0.3)	0	(0.0)	0.00
HPV 49	1	(0.2)	3	(0.6)	3	(0.8)	0	(0.0)	0.00
HPV 58	1	(0.2)	0	(0.0)	1	(0.3)	0	(0.0)	0.00
HPV 75	0	(0.0)	1	(0.2)	0	(0.0)	0	(0.0)	—
HPV 76	3	(0.7)	7	(1.5)	6	(1.6)	1	(0.3)	0.28
HPV 80	0	(0.0)	6	(1.3)	5	(1.4)	0	(0.0)	0.00
HPV 93	1	(0.2)	0	(0.0)	1	(0.3)	0	(0.0)	0.00
HPV 96	0	(0.0)	4	(0.9)	2	(0.5)	0	(0.0)	0.00
HPV 98	0	(0.0)	2	(0.4)	0	(0.0)	0	(0.0)	—
HPV 100	2	(0.4)	0	(0.0)	2	(0.5)	0	(0.0)	0.00
HPV 104	1	(0.2)	1	(0.2)	2	(0.5)	0	(0.0)	0.00
HPV 105	0	(0.0)	4	(0.9)	2	(0.5)	0	(0.0)	0.00
HPV 107	11	(2.4)	7	(1.5)	13	(3.5)	0	(0.0)	−0.01
HPV 110	7	(1.5)	12	(2.6)	14	(3.8)	0	(0.0)	−0.02
HPV 111	4	(0.9)	4	(0.9)	6	(1.6)	0	(0.0)	−0.01
HPV 113	4	(0.9)	5	(1.1)	8	(2.2)	0	(0.0)	−0.01
HPV 115	0	(0.0)	3	(0.6)	3	(0.8)	0	(0.0)	0.00
HPV 120	6	(1.3)	6	(1.3)	7	(1.9)	0	(0.0)	−0.01
HPV 122	2	(0.4)	2	(0.4)	3	(0.8)	1	(0.3)	0.50
HPV 124	0	(0.0)	8	(1.7)	5	(1.4)	0	(0.0)	0.00
HPV 143	1	(0.2)	1	(0.2)	2	(0.5)	0	(0.0)	0.00
HPV 145	1	(0.2)	2	(0.4)	2	(0.5)	0	(0.0)	0.00
HPV 151	2	(0.4)	3	(0.6)	3	(0.8)	0	(0.0)	0.00
Beta-1 species	16	(3.5)	45	(9.6)	36	(9.7)	2	(0.5)	0.06
Beta-2 species	60	(13.2)	67	(14.3)	88	(23.8)	5	(1.4)	−0.02
Beta-3 species	4	(0.9)	14	(3.0)	12	(3.2)	1	(0.3)	0.14
Beta-5 species	0	(0.0)	4	(0.9)	2	(0.5)	0	(0.0)	0.00

HPV: human papilloma virus; Kappa values: <0.0 = Poor, 0.00–0.20 = Slight, 0.21–0.40 = Fair, 0.41–0.60 = Moderate, 0.61–0.80 = Substantial and 0.81–1.00 = Almost perfect.

**Table 3:** Positivity of cutaneous gamma HPV types in the genital and anal samples among men who have sex with women (MSW).

HPV type	Sample site								
	Genital Number (%)		Anal Number (%)		Among those with both genital and anal samples tested				
					Positive on either site Number (%)		Positive on both sites Number (%)		Kappa
Individuals assessed	381		454		309		309		
Any HPV type	47	(12.3)	64	(14.1)	75	(24.3)	4	(1.3)	−0.03
HPV 4	5	(1.3)	5	(1.1)	8	(2.6)	0	(0.0)	−0.01
HPV 50	5	(1.3)	10	(2.2)	11	(3.6)	0	(0.0)	−0.02
HPV 88	1	(0.3)	0	(0.0)	0	(0.0)	0	(0.0)	—
HPV 95	5	(1.3)	8	(1.8)	9	(2.9)	0	(0.0)	−0.01
HPV 103	4	(1.0)	3	(0.7)	4	(1.3)	1	(0.3)	0.40
HPV 108	9	(2.4)	3	(0.7)	9	(2.9)	0	(0.0)	−0.01
HPV 109	0	(0.0)	2	(0.4)	1	(0.3)	0	(0.0)	0.00
HPV 112	0	(0.0)	1	(0.2)	1	(0.3)	0	(0.0)	0.00
HPV 116	0	(0.0)	1	(0.2)	1	(0.3)	0	(0.0)	0.00
HPV 121	10	(2.6)	7	(1.5)	14	(4.5)	0	(0.0)	−0.02
HPV 123	2	(0.5)	5	(1.1)	5	(1.6)	0	(0.0)	−0.01
HPV 127	1	(0.3)	0	(0.0)	1	(0.3)	0	(0.0)	0.00
HPV 128	2	(0.5)	1	(0.2)	2	(0.6)	0	(0.0)	0.00
HPV 129	0	(0.0)	1	(0.2)	1	(0.3)	0	(0.0)	0.00
HPV 130	1	(0.3)	2	(0.4)	2	(0.6)	0	(0.0)	0.00
HPV 132	2	(0.5)	10	(2.2)	11	(3.6)	0	(0.0)	−0.01
HPV 133	0	(0.0)	5	(1.1)	1	(0.3)	0	(0.0)	0.00
HPV 148	2	(0.5)	3	(0.7)	2	(0.6)	0	(0.0)	0.00
HPV 149	0	(0.0)	2	(0.4)	0	(0.0)	0	(0.0)	—
HPV 156	6	(1.6)	6	(1.3)	9	(2.9)	0	(0.0)	−0.01
HPV 158	0	(0.0)	1	(0.2)	0	(0.0)	0	(0.0)	—
HPV 159	1	(0.3)	0	(0.0)	0	(0.0)	0	(0.0)	—
Gamma-1 species	10	(2.6)	14	(3.1)	17	(5.5)	0	(0.0)	−0.03
Gamma-3 species	5	(1.3)	10	(2.2)	11	(3.6)	0	(0.0)	−0.02
Gamma-6 species	13	(3.4)	6	(1.3)	13	(4.2)	1	(0.3)	0.13
Gamma-7 species	2	(0.5)	8	(1.8)	5	(1.6)	0	(0.0)	−0.01
Gamma-8 species	0	(0.0)	1	(0.2)	1	(0.3)	0	(0.0)	0.00
Gamma-9 species	0	(0.0)	2	(0.4)	2	(0.6)	0	(0.0)	0.00
Gamma-10 species	10	(2.6)	14	(3.1)	16	(5.2)	0	(0.0)	−0.02
Gamma-12 species	4	(1.0)	13	(2.9)	13	(4.2)	0	(0.0)	−0.02
Gamma-13 species	2	(0.5)	1	(0.2)	2	(0.6)	0	(0.0)	0.00
Gamma-18 species	6	(1.6)	6	(1.3)	9	(2.9)	0	(0.0)	−0.01

HPV: human papilloma virus; Kappa values: <0.0 = Poor, 0.00–0.20 = Slight, 0.21–0.40 = Fair, 0.41–0.60 = Moderate, 0.61–0.80 = Substantial and 0.81–1.00 = Almost perfect.

For β-HPVs, 38 types were detected in participants of the total 43 β-HPVs. The most commonly-detected β-HPV types in the genital sites were β-HPV-22 and β-HPV-107 (each: n = 11, 2.4%), β-HPV-23 and β-HPV-38 (each: n = 10, 2.2%) and β-HPV-5 (n = 9, 2.0%). The most commonly-detected β-HPV types in the anal canal were β-HPV-22 (n = 14, 3.0%), and β-HPV-110 (n = 12, 2.6%), and β-HPV-5 and β-HPV-12 (each: n = 10, 2.1%) (Table 2). Multiple β-HPVs were found in 22.4% (17 of 76 β-HPV-positive men) and 29.0% (31/107) of the genital and anal sites, respectively (data not shown). The most commonly-detected species was β2-HPV in both (n = 60, 13.2% in genital and n = 67, 14.3% in anal) anatomical sites. Low overall (n = 13, 3.5%) and type-specific (n = 4, 1.1%) concordance for β-HPVs were observed between genital and anal samples (Table 2).

For γ-HPVs, 22 of the 29 screened types were detected. The most common γ-HPV types in genital samples were γ-HPV-121 (n = 10, 2.6%), γ-HPV-108 (n = 9, 2.4%) and γ-HPV-4, γ-HPV-50 and HPV-95 (each: n = 5, 1.3%), while γ-HPV-50 and γ-HPV-132 (each: n = 10, 2.3%), γ-HPV-95 (n = 8, 1.8%) and γ-HPV-121 (n = 7, 1.5%) were the most common types found in anal specimens (Table 3). Multiple γ-HPV infections were detected in 19.1% (9/47 γ-HPV-positive men) of genital and 25.0% (16/64) of anal samples (data not shown). The most commonly-detected γ HPV species were γ-6 in genital (n = 13, 3.4%) and γ-1 and γ-10 (each: n = 14, 3.1%) among sites (Table 3). Similar to β-HPVs, low overall (n = 4, 1.3%) and type-specific (n = 2, 0.6%) concordance for γ-HPVs was also observed between genital and anal samples (Table 3).

A multivariate analysis of effect of participant characteristic on β-HPVs (Table 4) and γ-HPVs (Table 5) in genital and anal samples among MSW was conducted. Interestingly, both β- and γ-HPV prevalence was observed to be higher although nonsignificant in the anal samples than in the genital samples.

**Table 4:** Adjusted analysis of effect of participant characteristic on cutaneous beta HPV types in genital and anal samples among men who have sex with women (MSW).

	Genital site			Anal site			Genital and anal sites combined*		
	No. assessed	No. HPV positive (%)	Adjusted odds ratio (95% CI)**	No. assessed	No. HPV positive (%)	Adjusted odds ratio (95% CI)**	No. assessed	No. HPV positive (%)	Adjusted odds ratio (95% CI)**
Participants	453	76 (16.8)		470	107 (22.8)		370	122 (33.0)	
Entry age (years)									
<25	88	20 (22.7)	1.0	88	13 (14.8)	1.0	74	26 (35.1)	1.0
25+	358	54 (15.1)	0.5 (0.3–1.0)	346	70 (20.2)	1.2 (0.6–2.4)	292	93 (31.8)	0.8 (0.5–1.3)
Age of sexual debut (years)									
<18	254	39 (15.4)	1.0	246	45 (18.3)	1.0	210	62 (29.5)	1.0
18+	193	35 (18.1)	1.4 (0.8–2.4)	190	39 (20.5)	1.3 (0.8–2.2)	157	58 (36.9)	1.7 (1.1–2.6)
No. of life-time sex partners									
1–9	135	23 (17.0)	1.0	124	16 (12.9)	1.0	110	33 (30.0)	1.0
10–19	106	18 (17.0)	1.1 (0.5–2.2)	102	18 (17.6)	1.6 (0.7–3.4)	85	26 (30.6)	1.2 (0.7–2.2)
20+	205	33 (16.1)	1.0 (0.5–1.9)	208	50 (24.0)	2.6 (1.3–5.1)	171	61 (35.7)	1.7 (1.0–2.8)
p for trend			0.865			0.006			0.042
Detected genital *C*. *trachomatis*									
Negative	418	70 (16.7)	1.0	430	103 (24.0)	1.0	339	113 (33.3)	1.0
Positive	25	4 (16.0)	0.9 (0.3–2.8)	29	4 (13.8)	0.6 (0.2–1.9)	23	8 (34.8)	1.0 (0.4–2.2)
Self-reported genital *C*. *trachomatis* in the past									
No	313	50 (16.0)	1.0	306	59 (19.3)	1.0	259	85 (32.8)	1.0
Yes	131	24 (18.3)	1.3 (0.7–2.2)	127	23 (18.1)	0.7 (0.4–1.3)	105	33 (31.4)	0.9 (0.6–1.4)

Footnote: HPV: human papilloma virus; CI: confidence interval; *Only individuals whose samples were tested for both sites were included in the regression model and HPV positivity is defined as positive on either genital or anal sites; **All variables included in the regression model.

**Table 5:** Adjusted analysis of effect of participant characteristic on cutaneous gamma HPV types in genital and anal samples among men who have sex with women (MSW).

	Genital site			Anal site			Genital and anal sites combined*		
	No. assessed	No. HPV positive (%)	Adjusted odds ratio (95% CI)**	No. assessed	No. HPV positive (%)	Adjusted odds ratio (95% CI)**	No. assessed	No. HPV positive (%)	Adjusted odds ratio (95% CI)**
Participants	381	47 (12.3)		454	64 (14.1)		309	75 (24.3)	
Entry age (years)									
<25	77	12 (15.6)	1.0	88	14 (15.9)	1.0	65	17 (26.2)	1.0
25+	302	35 (11.6)	0.8 (0.4–1.8)	331	35 (10.6)	0.5 (0.3–1.1)	243	57 (23.5)	1.0 (0.5–1.8)
Age of sexual debut (years)									
<18	219	29 (13.2)	1.0	234	30 (12.8)	1.0	176	44 (25.0)	1.0
18+	161	18 (11.2)	1.1 (0.6–2.3)	187	20 (10.7)	1.0 (0.5–2.0)	133	31 (23.3)	1.1 (0.6–1.8)
No. of life-time sex partners									
1–9	102	12 (11.8)	1.0	119	13 (10.9)	1.0	83	20 (24.1)	1.0
10–19	93	6 (6.5)	0.5 (0.2–1.5)	98	9 (9.2)	0.8 (0.3–2.1)	73	12 (16.4)	0.6 (0.3–1.3)
20+	184	29 (15.8)	1.6 (0.7–3.7)	202	28 (13.9)	1.4 (0.6–3.0)	152	43 (28.3)	1.4 (0.7–2.5)
Detected genital *C*. *trachomatis*									
Negative	340	37 (10.9)	1.0	414	63 (15.2)	1.0	271	66 (24.4)	1.0
Positive	30	9 (30.0)	3.4 (1.4–8.5)	28	1 (3.6)	0.2 (0.0–1.9)	27	8 (29.6)	1.5 (0.7–3.3)
Self-reported genital *C*. *trachomatis* in the past									
No	259	34 (13.1)	1.0	294	30 (10.2)	1.0	211	49 (23.2)	1.0
Yes	120	13 (10.8)	0.8 (0.4–1.7)	124	20 (16.1)	1.6 (0.8–3.0)	97	26 (26.8)	1.2 (0.7–1.9)

Footnote: HPV: human papilloma virus; CI: confidence interval; *Only individuals whose samples were tested for both sites were included in the regression model and HPV positivity is defined as positive on either genital or anal sites; **All variables included in the regression.

Having a sexual debut at the age of 18 years and above resulted in higher but nonsignificant β-HPV-positivity in both genital (ORs = 1.4, 95% CI: 0.8–2.4) and anal (ORs = 1.3 (95% CI: 0.8–2.2)) samples compared to those starting sexual activities below 18 years. The prevalence of anal β-HPVs increased with increasing number of lifetime sexual partners (p for trend: 0.006 for anal β-HPVs and 0.865 for genital β-HPVs); this association was strongest among the men with more than 20 lifetime sexual partners (OR 2.6, 95% CI: 1.3–5.1). For the γ-HPV types, no linear trend was observed in the genial or anal samples.

The detection of β-HPVs in genital samples was also slightly associated with present genital *C*. *trachomatis* infection (OR = 1.3, 95% CI: 0.7–2.2). Regarding γ-HPVs, only self-reported genital *C*. *trachomatis* infection in the past was associated with an increased anal γ-HPV-positivity (OR = 1.6, 95% CI 0.9–3.0).

Due to the lack of genital samples from the HIV-positive men, no comparison was possible for HIV status in genital samples. Among the anal sites, both cutaneous HPV genera were more commonly detected in HIV-positive men than HIV-negative men (crude OR = 12.2, 95% CI: 5.3–28.1 for β-HPVs and crude OR = 7.2, 95% CI: 3.3–15.4 for γ-HPVs, respectively) (Table 6).

**Table 6:** Crude analysis of the effect of HIV status on cutaneous beta and gamma HPV types in anal samples among men who have sex with women (MSW).

	No. assessed	No. HPV positive (%)	Crude odds ratio (95% CI)
***For beta HPV types***			
Participants	470	107	
HIV status			
Negative	439	84 (19.1)	1.0
Positive	31	23 (74.2)	12.2 (5.3–28.1)
***For gamma HPV types***			
Participants	454	64	
HIV status			
Negative	423	49 (11.6)	1.0
Positive	31	15 (48.4)	7.2 (3.3–15.4)

HPV: human papilloma virus; HIV: human immune-deficiency virus.

Forty-seven (7.9%) recently diagnosed STIs were observed in the study, including HIV in one (0.2%), *N*. *gonorrhea* in one (0.2%), *C*. *trachomatis* in 36 (6.1%), herpes simplex virus in 3 (0.5%) and *M*. *genitalium* in 7 (1.2%) men, respectively (data not shown). The evaluation of patients with the National Institutes of Health Chronic Prostatitis Symptom Index (NIH-CPSI)^31^ observed no association between the clinical symptoms and the prevalence of cutaneous HPVs in the anal and genital area (data not shown).

## Discussion

This is the first report of the determinants for anal and genital presence of a broad range of both β- and γ-HPV types in a cohort of 554 Russian heterosexual men investigated and compared. Importantly, despite the abundance of both HPV genera observed in the anogenital area in the current study, the type-concordant association between genital and anal sites was uncommon. Although some association was observed between the number of lifetime sex partners and genital and anal prevalence for either genera (in particular for β-HPVs), poor type-specific positive concordance between the two sites and higher but nonsignificant prevalence of both HPV genera in anal sites compared to genital samples provides more evidence to the existence of such transmission route as autoinoculation, which needs to be explored further.

Importantly, a substantially higher prevalence of β- and γ-HPVs was detected in HIV-positive than HIV-negative men. No significant association between the anal prevalence of the two cutaneous genera with HIV status or sexual behavioral factors were observed in a study among Italian MSM, applying the same diagnostic test^27^. The current study supports the evidence that impairment of the host’s immune surveillance may impact β- and γ-HPV infections differently^28^. Further studies should be extended with genital and non-anogential sites, to better understand the association between HIV infection status and subsequent HPV acquisition and vice versa.

To our knowledge, the only study that investigated the prevalence of two cutaneous genera in anal and genital specimens obtained among MSW was the HIM study. First, the study reported the high prevalence of β- and γ-HPVs in the anogenital skin by sequencing 25 β- and 3 γ-HPVs in the anal canal in 164 MSW^29^ from the USA, Brazil and Mexico. As the epithelium of male genitals is known to be rich with a broad range of HPVs^20^, the current study employed the proficient^32^ Luminex assay, which allowed for the detection of the broadest range of β- and γ-HPV types and compared genital and anal prevalence within the same individuals. Applying the Luminex assay, the study from the Western hemisphere assessed diversity of β-HPV types at oral gargles, anal canal and genital sites specimens obtained from 717 men^21^. The current study adds to this new evidence by including 554 MSW, the first such known sample in the Eastern hemisphere.

Another strength of the current study was the use of combined penile and urethral samples in the detection of cutaneous HPVs. Penile and urethral swabs have been found more likely to have the highest prevalence with α-HPV infections^33^. The addition of urine samples to penile swabs in the detection of HPV in men has illustrated how useful the combination of genital samples could be for epidemiological or clearance studies^34^. The combination of the external genital and scrotum swabs was also employed in the detection of β-HPV types in the HIM study^21^.

The obtained data also do not represent the general population. The study limitations also include a low number of MSM observations, as research on MSM populations in Russia remains delicate^35^ and the lack of retrospective genital samples obtained from the HIV-positive men, as only anal and serum samples were primary collected^36^ during the survey on Chlamydia LGV infection. Larger, preferably prospective, studies to further explore these issues would ideally include both penile and urethral specimens to elucidate the role of immunosuppression on the prevalence of genital β- and γ-HPVs among HIV-positive and HIV-negative MSW and MSM. To help in understanding the potential transmission routes of cutaneous HPVs, further behavioral studies should also include more detailed surveys on sexual behaviors.

The routes for the transmission of HPVs into the anal canal in MSW are not clear. Minimizing the risk of potential contamination from the anatomical sites close to the anal verge at the time of anal sampling, the procedure should be performed by a single trained physician in a standard manner, as in the present study. The presence of cutaneous HPV DNA in the anogenital area may reflect deposition of virions released from other body sites with productive infections^20^. In this respect, self-inoculation or transmission from skin, mucosa or secretions from the partners may result in the detection of β- and γ-HPV DNAs in the anal canal of men. Identical cutaneous β-HPV types in both penile and anal intraepithelial neoplasia were found in a study on HIV-positive MSM, assuming the dissemination between different anogenital regions within a given patient^25^. In a study among 25 heterosexual couples, the man’s hand either β- or γ-HPV types were found in the anogenitals of the female partner in 25% of the visits, while the woman’s hand β- and γ-HPV types were found in the males’ anogenital area in approximately 50% and 25% of the visits, respectively^30^.

In the HIM Study, multiple β-HPV types were more likely to be detected at the genital than at the anal canal^21^. In the current study, next-to-nothing type-specific concordance between genital and anal sites was observed for both HPV genera in HIV-negative MSW. It could also be envisioned that different transmission routes may result in the discrepancy on HPV prevalence between different sites, regardless their sexual practices. In the studies of heterosexual couples, transmission of α-HPVs between hands and genitals or apparent self-inoculation (primarily in men) events^37^ resulted in modest concordance rates between genital and non-genital sampled sites^38^. The further studies on extended number of anatomical sites may provide the answer.

Similar to others^39^, the current study could not distinguish new or persistent HPV infection. Detecting HPV transcripts could be seen as a useful options, since the detection of HPV DNA may be the result of transient deposition^39^. The presence of HPV DNA does not necessarily indicate presence of infectious virus^18,40–42^, although the relatively high rate of concordance of β- and γ-HPVs between sex partners found in the recent study of 25 couples has suggested that the anogenitals represents a more common area for infectivity than previous thought and that sexual transmission is possible^30^. β-HPV does seem to exhibit activities that can promote oncogenesis, although using distinct from α-HPV mechanisms such as cell transformation promotion and deregulation of pathways linked to the host immune response^43–45^. In addition, recently reported associations with the risk of incident head and neck cancer others than α-HPV types, which included γ11- and γ12-HPV species and β1-HPV-5 type^46^, advocates the needs for further, preferably prospective, studies on etiological role of cutaneous HPVs in carcinogenesis.

In summary, the current study extends our knowledge regarding β- and γ-HPV types and their distribution in diverse male populations, which is essential for developing a better understanding of the natural history, transmission dynamics, and the potential role of different HPV types as co-factors in the development of anogenital malignancies.

## Methods

### Study population

The study settings and methods have been described in detail elsewhere^22^. Genital and anal samples originally obtained from 609 men in two clinic-based studies in St. Petersburg, Russia were available for testing. The first was a pilot case-control study investigating anal Chlamydia LGV infection performed in an infectious disease hospital providing treatment for HIV-infected, from December 2005 to January 2006^36^. The second was conducted among men seeking routine STI testing at the urology units of two university outpatient clinics, from February 2006 to February 2009^47^.

For both studies, men were eligible for enrolment if they were at least 18 years old and reported no anorectal disorders. A medical history and a standard physical examination were completed. Participants completed a questionnaire concerning sexual behaviour (age at enrolment, age at the time of sexual debut, number of lifetime sex partners, and sexual preferences). Data on age and sexual preferences were only available from the infectious disease hospital participants.

All men self-reported their sexual preferences. If a man reported having had sex (anal or oral) with at least one other man during his lifetime, he was categorized as MSM. If only sex with women was reported, a man was classified as MSW. The questionnaire was administered during a face-to-face interview, before anal sampling. Importantly for the local context, all participants were informed that this information would be unavailable for the third part. In addition, the responses were coded^22^.

**Figure 1:**
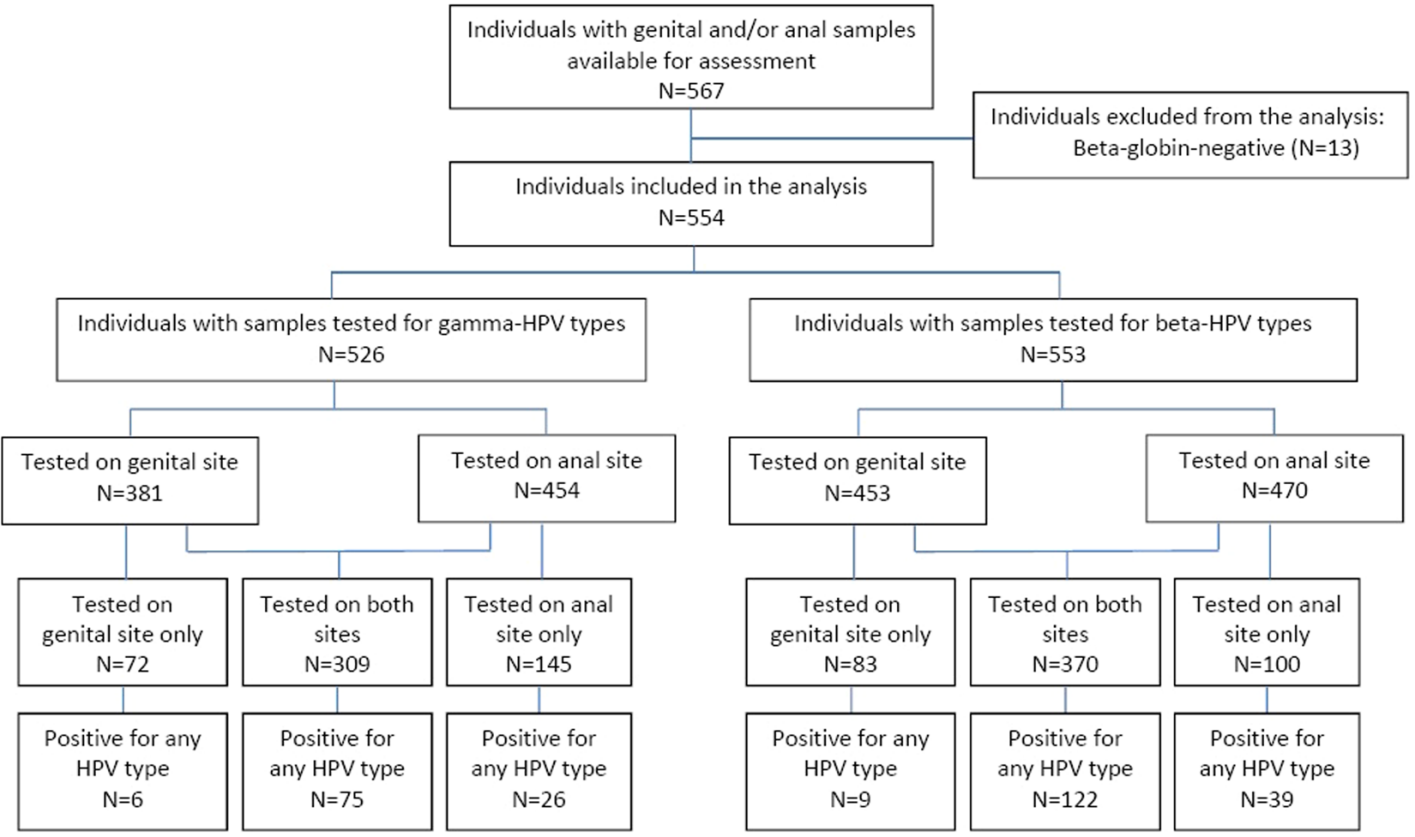
Flow chart of individuals whose genital and anal samples were tested for beta- and gamma-HPV types.

After excluding samples from 40 MSM (6.6%) and β-globin-negative samples from 13 (out of 569) individuals (2.3%), in total the genital and anal samples obtained from 554 self-reported as MSW were included in the analysis (Fig. 1).

All participants from the urology units provided blood samples for HIV testing. All previously-tested HIV-positive patients were receiving HAART therapy at the time of enrolment.

All participants provided written informed consent for the relevant study. Institutional review boards approved the studies, the Department of Clinical Investigations and Intellectual Property, of St. Petersburg Medical Academy of Postgraduate Studies (North-Western State Medical University named after I.I. Mechnikov since 2011) under the Federal Agency of Public Health and Social Development of Roszdrav.

DNA samples and questionnaire data were anonymized, and no study personnel besides the principal investigator had access to participants’ identifying information.

### Collection of samples and DNA extraction

Samples from genital sites and anal canal were collected. To minimize the risk of potential contamination from the anatomical sites close to the anal verge at the time of anal sampling, the procedure was performed by a single trained physician in a standard manner. Before sampling, men were instructed to abstain from any form of sex for 3–5 days and from urination for 3–4 hours. Urethral sampling was performed as described elsewhere^47^. The study clinician first sampled the distal urethra (inserted up to about 2 cm inside) followed by the penis (including the coronal sulcus, glans penis and the penile shaft) using two separate brushes. The swabs were rinsed in 1000 µl of phosphate buffer in two separate tubes. The anal canal was sampled with a third brush wetted with phosphate buffered saline, inserted about 2 cm into the anal canal and rotated 360 degree clockwise and anticlockwise directions. The swab was then rinsed in 1000 µl of phosphate buffered saline in a separate tube. The tubes were placed in a refrigerator at 4 °C. The samples were then transferred to a −20 °C freezer and stored until HPV testing.

Using a 100 µl aliquot of the original anal sample, DNA was extracted in the laboratory of the Department of Laboratory Medicine at Karolinska Institutet (Stockholm, Sweden), using a freeze-thaw-boil procedure, as previously described^48^. The DNA samples were shipped to the laboratory of the Group of Infections and Cancer Biology at IARC (Lyon, France) for β- and γ-HPV-specific genotyping.

### Detection and typing of HPV

Genital and anal samples were tested for the presence of HPVs using type-specific PCR bead-based multiplex genotyping (TS-MPG) assays that combine multiplex polymerase chain reaction (PCR) and bead-based Luminex technology (Luminex Corp., Austin, TX, USA), as described elsewhere^21,49–53^. The multiplex type-specific PCR method uses specific primers for the detection of 43 β-HPVs (species β-1: 5, 8, 12, 14, 19, 20, 21, 24, 25, 36, 47, 93; β-2: 9, 15, 17, 22, 23, 37, 38, 80, 100, 104, 107, 110, 111, 113, 120, 122, 145, 151; β-3: 49, 75, 76, 115; β-4: 92; β-5: 96, 150)^21^ and 29 γ-HPVs (species γ-1: 4, 65, 95; γ-2: 48; γ-3: 50; γ-4: 156; γ-5: 60, 88; γ-6: 101, 103, 108; γ-7: 109, 123, 134, 149; γ-8: 112, 119; γ-9: 116, 129; γ-10: 121, 130, 133; γ-11: 126; γ-12: 127, 132, 148; γ-13: 128; γ-14: 131; and HPV-SD2^54^). HPV type species were classified according to de Villiers^3^. Two primers for the amplification of the β-globin gene were included to provide a positive control for the quality of the DNA in the sample^55^.

In the current study, 10 μl of the anal sample and 10 μl of the genital samples were analysed. A 10 μl volume of genital sample was obtained by combining into one sample 5 μl of penile and 5 μl of urethral aliquots. Following multiplex PCR amplification, 10 μl of each reaction mixtures were analysed by multiplex genotyping using the Luminex technology^49,52^.

### Statistical analysis

In the current analyses we included only individuals, who had both genital and anal samples, whose samples were tested as beta-globin-positive. Paired genital and anal samples were collected in urology participants in parallel. For HIV-positive patients, only anal samples were obtained.

The prevalence (overall, type- and species-specific) of β- and γ-HPVs by anatomical sites was presented as proportions. “Type-specific positive concordance” was defined as having the same HPV genotype in both genital and anal samples of a participant, with the Kappa-based extent of agreement between the two anatomical sites measured (Kappa values: <0.0 poor, 0.00–0.20 slight, 0.21–0.40 fair, 0.41–0.60 moderate, 0.61–0.80 substantial and 0.81–1.00 almost perfect)^56^. Odds Ratios (OR) and their 95% confidence intervals (CI) were calculated using logistic regression to assess associations between β- and γ-HPV positivity and age, age at sexual debut, number of lifetime sexual partners, past and present *C*. *trachomatis* infection (all categorical). Except HIV status (when crude analysis was performed only), all variables were included in the adjusted regression model. All analyses were completed using Stata versions 14 (StataCorp, College Station, TX, USA).

### Ethical approval

Ethical Committee of the Department of Clinical Investigations and Intellectual Property of St. Petersburg Medical Academy of Postgraduate Studies (North-Western State Medical University named after I.I. Mechnikov since 2011) under the Federal Agency of Public Health and Social Development of Roszdrav (Extract from Minutes No. 10 of SPbMAPS Ethical Committee meeting; date of approval: 10 November 2010).
